# Evaluation of tumor microenvironmental immune regulation and prognostic in lung adenocarcinoma from the perspective of purinergic receptor P2Y13

**DOI:** 10.1080/21655979.2021.1971029

**Published:** 2021-09-08

**Authors:** Jiangtao Wang, Weiwei Shi, Yandong Miao, Jian Gan, Quanlin Guan, Juntao Ran

**Affiliations:** a Department of Radiation Oncology, The First Hospital of Lanzhou University, Lanzhou, Gansu, PR China; b The First Clinical Medical College of Lanzhou University, Lanzhou, PR China; c Clinical Skills Center of Yantai Affiliated Hospital of Binzhou Medical College, Yantai, PR China; d Department of Oncology Surgery, The First Hospital of Lanzhou University, Lanzhou, Gansu, PR China

**Keywords:** P2RY13, tumor microenvironmental, immune regulation, lung adenocarcinoma, prognosis

## Abstract

Tumor-infiltrating immune cells (TICs) can serve as an important indicator to evaluate the prognosis and therapeutic response in lung adenocarcinoma (LUAD). The identification of mutated genes that can affect the abundance of TICs and prognosis has practical implications. In the presented study, tumor microenvironment (TME) scoring was performed by the ESTIMATE scoring system on 598 RNA transcripts selected from the TCGA database to determine the proportions of immune cells and stromal cells. The infiltration difference of TICs in LUAD samples was obtained by CIBERSORT. The ‘immuneeconv’ R software package, which integrates six latest algorithms, including TIMER, xCell, MCP-counter, CIBERSORT, EPIC and quanTIseq were used to verify the correlation between purinergic receptor P2Y13 (*P2RY13*) and immune cells. Based on RNA sequencing analysis of the Lewis lung cancer-bearing model in C57BL/6 mice and immunohistochemistry (IHC) of human LUAD tissues, the expression of *P2RY13* and associated pathways were verified. It was shown that differentially expressed genes (DEGs) obtained by interactive analysis based on Immunescore and Stromalscore were significantly enriched in immune-related pathways. The expression of *P2RY13* was significantly associated with prognosis and clinicopathological characteristics of LUAD patients. More importantly, this gene played an important role in maintaining the immune dominant environment and changing the regulation of TICs. *P2RY13* expression was positively correlated with the infiltration of dendritic cells (DCs) in various of tumor tissues as validated by the PanglaoDB scRNA-seq database. Therefore, *P2RY13* is expected to be a potential biomarker for predicting TME and the prognosis of LUAD after verification.

## GRAPHICAL ABSTRACT

**Figure:**
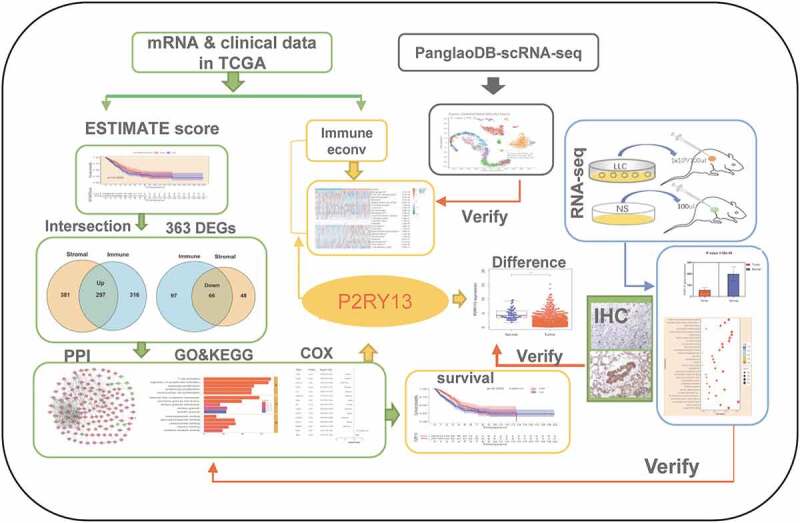

## Introduction

Lung cancer is the leading cause of cancer-related deaths worldwide [1], with an estimated 1.6 million deaths each year, which has become an important disease threatening human health [2]. 82% of which are directly caused by smoking, meaning that there will be approximately 107,870 lung cancer deaths attributed to smoking in 2021 [3]. Non-small cell lung cancer (NSCLC) accounts for more than 80% of all lung cancer, with lung adenocarcinoma (LUAD) being the most common [4]. The lower lung cancer survival rate reflects the high proportion of patients diagnosed with metastatic disease (57%), which has a 5-year relative survival rate of 6% [5]. With the continuous improvement of the level of comprehensive treatment especially immunotherapy, the prognosis of LUAD has gradually improved but remains at about 20% [6,7]. Therefore, there is an urgent need to improve the treatment strategy to increase the clinical benefit rate of LUAD.

Tumor progression is closely related to the tumor microenvironment (TME) [8]. Tumor cells promote angiogenesis by releasing extracellular signals and induce the recruitment of peripheral immune cells [9,10]. TME involving stromal cells and immune cells has a dual role as an immune editor, producing cytokines and performing immunomodulatory functions, thus directly or indirectly promoting or inhibiting the growth and metastasis of tumor cells [11]. Although previous studies on the effect of tumor-infiltrating immune cells (TICs) in TME on tumor progression and prognosis are scarce, an increasing number of studies have shown that TICs can be used as an effective indicator of prognosis and therapeutic efficacy [12–15]. Therefore, the scoring system based on immune cells and stromal cells is particularly important for judging dynamic regulation in TME and improving immunotherapy strategies [16,17]. Although the mechanisms involved have not been fully elucidated, TME has been considered as a target for various tumor immunotherapies. Among them, inhibitors targeting immune checkpoints have become the first or second-line treatment for NSCLC [18]. For example, antibodies targeting programmed cell death 1 receptor (PD-1) or ligand (PD-L1) can activate the anti-tumor effect of cytotoxic T lymphocytes [19]. Unfortunately, the effectiveness of immunotherapy can be reduced or altered by mutations in oncogenes. EGFR (epidermal growth factor receptor) mutations or ALK (anaplastic lymphoma kinase) rearrangements in NSCLC usually exhibit poor clinical benefits to checkpoint inhibitor (CPI) [20,21], although few reports have explained the underlying mechanism.

Considering this condition, the present study aimed to screen for mutated genes capable of assessing the immune microenvironment and prognosis of LUAD and thus provided targets for timely treatment or intervention. In this study, we performed the CIBERSORT immune score on RNA transcript data of LUAD downloaded from the TCGA database and screened for mutant genes significantly associated with the prognosis and TME of LUAD. The plausibility and reliability were then verified by RNA-seq and immunohistochemistry. The R package ‘immuneeconv’ was used to verify the correlation between the selected prognostic core genes and major immune infiltrating cells, which in turn provided preliminary data for future experimental verification and clinical analysis.

## Methods and materials

### Data retrieval and processing

In this research, we downloaded the RNA transcriptional sequence and clinicopathological information of LUAD from the TCGA database (https://tcga-data.nci.nih.gov/tcga/). The matrix file of RNA-seq for different samples was collected and annotated onto the genome. The expression of mRNA extracted from the matrix file obtained from the RNA-seq data, which containing 539 LUAD tissues and 59 adjacent non-tumorous tissue samples, and 535 clinical cases as of June 2020. Also, 1368 single-cell RNA-sequencing (scRNA-seq) datasets from the PanglaoDB database [22](https://panglaodb.se/search.html) were used to find cell types where the *P2RY13* gene was expressed. Besides, the TIMER2.0 database (http://timer.cistrome.org/) was used to further verify the correlation between *P2RY13* and immune cells. These data were obtained in strict accordance with the publication guidelines approved by the above database. Therefore, there was no requirement for ethics committee approval.

### Survival analysis based on Immunescore, Stromalscore, and Estimatescore

The immune-stromal components in the TME of LUAD samples were evaluated by the ‘ESTATE’ algorithm in the R package, and the corresponding Immunescore, Stromalscore, and Estimatescore were obtained, reflecting the proportion of immune cells, stromal cells, and the sum of both in the TME, respectively. The higher the score, the greater the proportion of the corresponding cells. The median score was selected as the cutoff value for the LUAD dichotomy, thus dividing respective patients into high-and low-risk groups. Survival analysis was performed using the Kaplan-Meier method and log-rank test. The survival information of each sample was obtained from the TCGA database, and the correlation between scores and overall survival (OS) was further analyzed using the R-package ‘survminer’ and ‘survival’. Then, according to the corresponding relationship between the scores and survival status, we obtained the survival curves with the Kaplan–Meier method based on the TME scores determined by the ESTIMATE scoring system, and p < 0.05 was considered statistically different by the log-rank test.

### Enrichment analysis and immune-related pathways construction based on differentially expressed genes (DEGs)

Based on the median scores of the Immunescore and Stromalscore, 598 samples were allocated to a high and low subgroup. Data extraction and integration were performed via Perl Script. Screening of DEGs was performed with R software (version 3.6.1), the ‘limma’ package. |Log (fold change, FC) |>1 and false-positive discovery (FDR, adjusted P-value) < 0.05 were set as the cutoffs. The functional annotation of Gene Ontology (GO) was performed using the R package ‘enrichplot’ (http://www.bioconductor.org/). The same method was applied for a Kyoto Encyclopedia of Genes and Genomes (KEGG) pathway enrichment analysis. A P-value of < 0.05 was considered statistically significant.

### Construction of protein-protein interaction (PPI) network based on the co-expressed genes screened from Immenescore and Stromalscore

The STRING database (http://www.string-db.org/) was used to construct the PPI network for the co-expressed genes (up-or down-regulated genes) screened by the Immunescore and Stromalscore, and reconstructed according to Cytoscape software version 3.6.1 to obtain hub genes. Among them, nodes with a minimum confidence interval greater than 0.9 were used to construct the interaction network by the MCODE tool [23].

### Identification of core genes related to prognosis based on Cox analysis

Univariate Cox analysis was used to identify LUAD prognosis-related genes, and the core genes selected by the PPI network were subjected to interactive analysis to obtain prognosis-related core genes. 95% confidence interval for each statistical analysis and a P-value<0.05 was considered statistically significant. The Human Protein Atlas online database (HPA, http://www.proteinatlas.org/) was used to verify the differential expression of CCR2 and P2RY13 in LUAD tumors and normal tissues. Besides, R-package ‘survminer’ and ‘survival’ were used to describe the relationship between *P2RY13* expression and OS. The Kaplan-Meier Plotter online database (http://kmplot.com/analysis/), whose resources include GEO, EGA, and TCGA, was used to verify the rationality of the *P2RY13* survival analysis we constructed. To verify whether *P2RY13* is an independent prognostic factor for LUAD, RNA-seq data downloaded from TCGA in HTSeq-FPKM format and clinical information were analyzed with the Cox regression module in the R package ‘survival’.

### Cell lines and cell culture

Mouse Lewis lung cancer cells (LLC) were purchased from the Cell Bank, Type Culture Collection, Chinese Academy of Sciences (CBTCCCAS). Cells were cultured in Dulbecco’s Modified Eagle Medium (GIBCO, US) containing 10% fetal bovine serum (BIOWEST, France) and 1% penicillin/streptomycin (HyClone) and were incubated at 37°C in 5% CO_2_.

### Mouse model

16 Six-week-old female C57BL/6 mice were purchased from Beijing Weitonglihua Laboratory Animal Technology Co., Ltd. (certificate number: 20201224Abzz0619000903). These mice were congenic C57BL/6 (backcrossed for five generations) and were then inbred. All animals were housed in specific pathogen-free (SPF) facilities (temperature 18–25°C, 40–60% humidity, 12-hour light and dark cycle) with free access to standard laboratory chow and water. To minimize potential confounders, we chose mice born in the same litter, and the mice used in the experiment had similar body weights. After one week of acclimatization, a computer random sequence generator was used to randomly select six mice as the control group (n = 6). Taking into account the risk of tumor-bearing failure, the initial number of tumor-bearing model group (experimental group) was set at 10 (n = 10), and the individual mouse was considered the experimental unit within the study. We chose a small sample size based on our pre-experiment results, 6–8 mice per group showed significant statistical differences. To establish the Lewis tumor-bearing model, mice were anesthetized by giving 1% sodium pentobarbital (80 mg/kg, intraperitoneal injection) before all operations. LLC cells (1x10^6^/100ul) were injected into the right oblique ribs of mice in the experimental group. Similarly, normal saline (NS, 100ul) was injected into the right flank of the control mice. The tumor group formed tumors in about 9–10 days from the inoculation, and from the 17th to the 20th day, samples were taken and submitted for RNA-seq (the tumor size should not exceed 1000mm^3^, the outcome index). Finally, cervical dislocation was used for euthanizing mice. In alleviating suffering, all mice were treated humanely. All experimental protocols for animal tumor models have been approved by the Ethics Committee of the First Hospital of Lanzhou University (approval number: LDYYLL2019-130). In the experimental group, six mice with tumor-bearing models were successfully constructed and continued to be included in the study, and four mice were excluded for failure or unsatisfactory tumor-bearing. In this experiment, we strictly controlled the use of experimental animals, so there were no extra surviving mice. Four unsuccessful tumor-bearing mice were euthanized at the same time as other mice, so no materials and testing were performed on them.

### Verification of P2RY13 expression based on RNA-seq of C57BL/6 mouse LLC tissue and immunohistochemistry (IHC) of human LUAD tissue

#### RNA-seq analysis

RNA-seq was performed on six tumors and normal tissues of C57BL/6 female mice respectively (relying on Shanghai Biotree Biotechnology Co., Ltd.), and the gene expression differences were analyzed based on RNA-seq data. Trend analysis and correlation analysis of gene expression in the tumor-bearing model and control group were performed using the R package. Besides, KEGG enrichment analysis was performed according to the DEGs.

#### Immunohistochemical analysis

A total of eight patients with histopathologically confirmed LUAD were enrolled in the IHC analysis. This study was approved by the Institutional Review Board of the First Hospital of Lanzhou University. IHC assays were performed using the horseradish peroxidase (HRP) method. Antibodies used were as following: the primary antibody used in this experiment was the P2Y13 polyclonal antibody from Thermo Fisher Scientific, catalog # PA5-111,286, RRID AB_2856696 (1:500 dilution, Thermo Fisher Scientific, Waltham, MA USA), the MAXVision TM HRP (Maxin, Fuzhou, China) as a secondary antibody. The positive expression of P2RY13 protein was located on the cell membrane. The relative staining intensity was defined as negative for <5%; weak (+) for 5–25%; moderate (++) for 25–50%, and strong (+++) for >50% of the tumor cells stained positive for P2RY13.

### Correlation and difference analysis of TICs

To analyze the correlation between the *P2RY13* and different immune cells, we performed Spearman correlation analysis using the R package ‘limma’, ‘ggplot2’, ‘ggpubr’, and ‘ggExtra’, with a pFilter of 0.05. For reliable immune score evaluation and the TICs demeanor distribution in LUAD tumor samples, we used the ‘immuneeconv’ R package, which integrates six latest algorithms, including TIMER, xCell, MCP-counter, CIBERSORT, EPIC, and quanTIseq. P < 0.05 were selected as the screening condition and the samples with a missing expression of immune cells were excluded. Systematic benchmarking of these algorithms was performed and each algorithm was found to have its unique performance and advantages. Selected samples were analyzed to determine the correlation between different immune cells and specific genes. This study referred to the Spearman correlation standard, which is defined as the Spearman correlation coefficient between variable levels, which varies between −1 and 1, where −1 is a negative correlation, 0 is no correlation and 1 is a positive correlation. *SIGLEC15, IDO1, CD274, HAVCR2, PDCD1, CTLA4, LAG3*, and *PDCD1LG2* are transcripts associated with immune checkpoints. The expression values of these eight genes were extracted to observe the differential distribution of immune checkpoint-associated genes in the high and low expression groups of *P2RY13*. All the above analysis methods and R package were implemented by R foundation for statistical computing (2020) version 4.0.3 and software packages ‘ggplot2’ and ‘pheatmap’.

## Results

Identification of a mutated gene with both immune microenvironment and prognostic functions is essential for improving immunotherapy and providing therapeutic targets in LUAD. In this study, the *P2RY13* gene obtained based on TME score and multiple biometric analysis is expected to become a target gene with the above characteristics. The expression of *P2RY13* and related pathways were verified by RNA-seq and immunohistochemistry based on the Lewis tumor-bearing model of C57BL/6 mice. The relevance of *P2RY13* to the infiltration of major immune cells in the LUAD immune microenvironment was explored based on the ‘immuneeconv’ R package and verified by the Panglao scRNA-seq database.

### The correlation between TME score and survival of patients with LUAD

**Figure 1.**
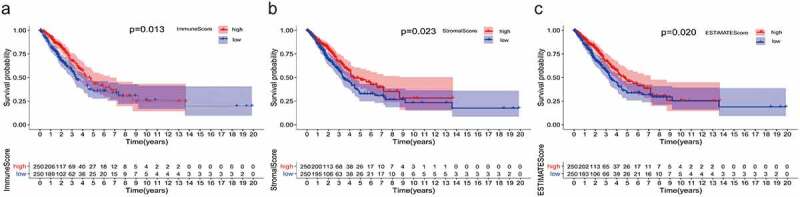
Correlation between TME scores and survival of patients with LUAD. (a) Kaplan–Meier survival curve for ImmuneScore with high or low score determined by the comparison with the median (*P* = 0.013 by log-rank test). (b) The survival curve for StromalScore with Kaplan–Meier method (*P* = 0.023 by log-rank test). (c) Kaplan–Meier survival curve for ESTIMATEScore (*P* = 0.020 by log-rank test). TME: Tumor microenvironment; LUAD: Lung adenocarcinoma

The immune-stromal components of LUAD transcriptional group information were scored by ‘limma’ and ‘estimate’ software packages, from which the Immunescore, Stromalscore, and Estimatescore were obtained. After further integration with the survival data after clinical information collation, the correlation between TME score and survival status of LUAD patients was obtained. As we predicted, the TME score, especially the Immunescore, was significantly correlated with the prognosis (Figure 1a-c). The higher the score, the longer the survival time.

### Screening and enrichment analysis of DEGs in LUAD based on the intersection of immunescore and stromalscore

**Figure 2.**
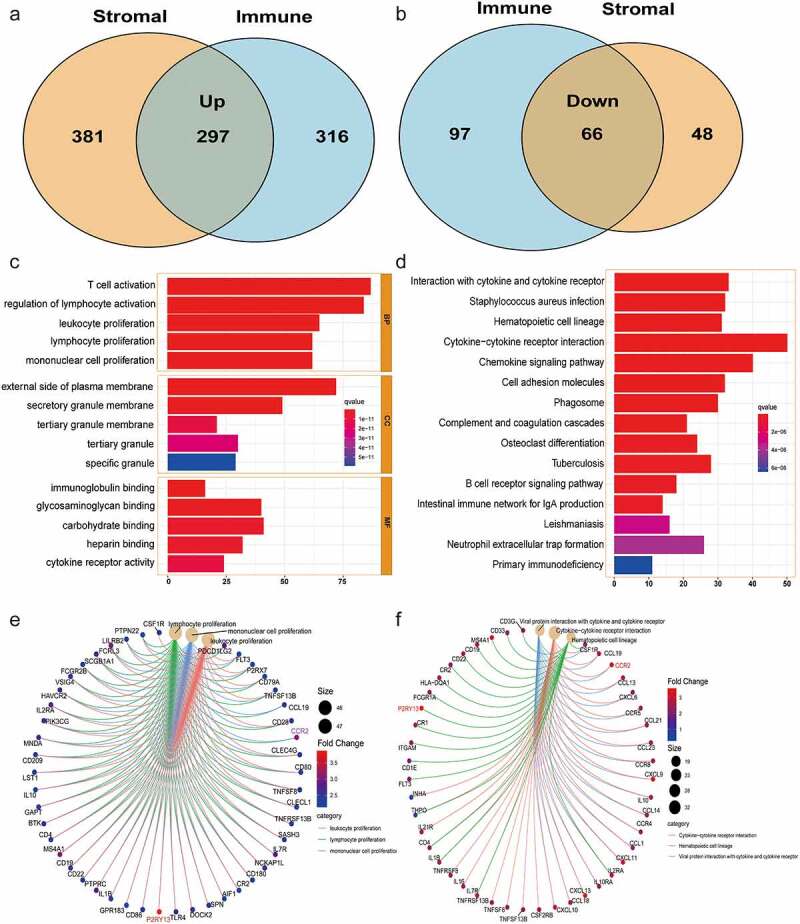
Venn plots, and enrichment analysis of GO and KEGG for DEGs based on ImmuneScore and StromalScore. (a-b) Venn plots showing the up-regulated and down-regulated DEGs shared by the ImmuneScore and StromalScore. (c-d) GO and KEGG enrichment analysis for 363 DEGs (**barplot**), terms with *p* and *q* < 0.05 were believed to be enriched significantly. (e-f) GO and KEGG enrichment analysis for 363 DEGs (**circos**). GO: Gene Ontology; KEGG: Kyoto Encyclopedia of Genes and Genomes; DEGs: differentially expressed genes

To further determine the changes in expression of immune and stromal cells gene profiles in TME, we performed a comparative analysis on the high and low score samples with the immune and stromal scores. A total of 776 DEGs were screened from the Immunescore, including 613 up-regulated genes and 163 down-regulated genes (Figure S1a). Similarly, 792 DEGs, including 678 up-regulated genes and 114 down-regulated genes, were obtained from the Stromalscore (Figure S1b). Venn plot showed that there were 297 co-expressed up-regulated genes and 66 co-expressed down-regulated genes in Immunescore and Stromalscore (Figure 2a-b), which were considered to be immune-related and significantly correlated with immune cell infiltration in LUAD. Based on this, we further performed GO and KEGG enrichment analysis on these DEGs (Figure 2c-f, Figure S2-3). Interestingly, these DEGs were mainly enriched in immune-related pathways including activated lymphocytes, immune response-activated cell surface receptor signal pathways, and interactions mediated by cytokines or chemokines.

### Screening of the prognostic-related genes regulating TME based on the intersection analysis of PPI network and Cox regression

**Figure 3.**
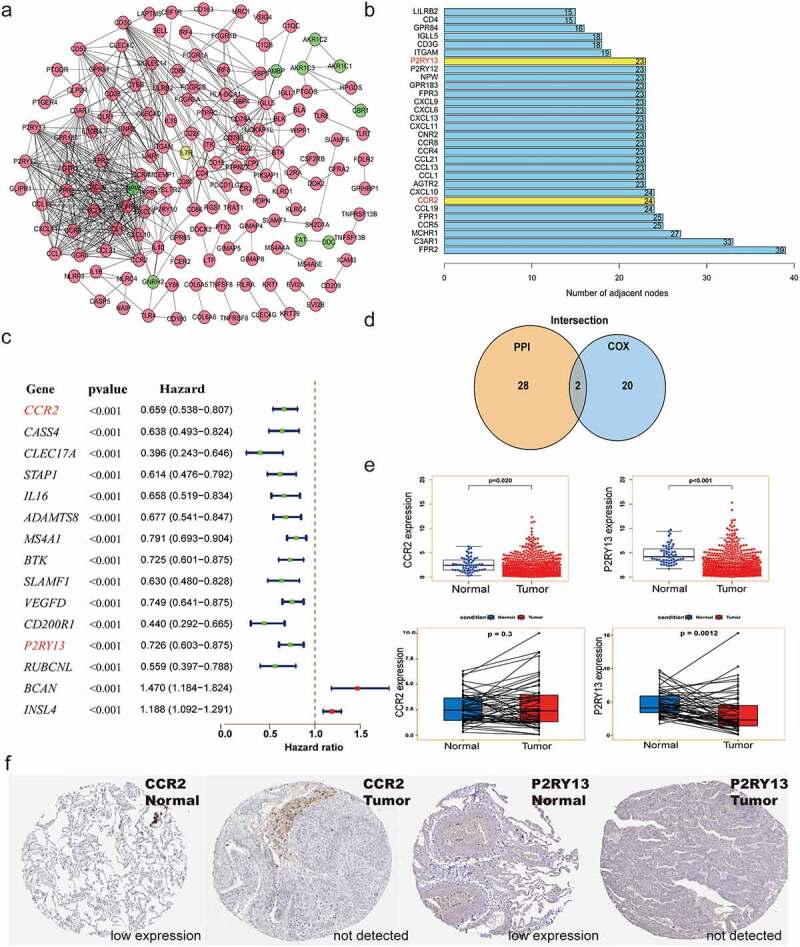
Screening of the key genes regulating TME based on the intersection analysis of PPI network and univariate Cox regression. (a) PPI network constructed with the minimum confidence intervals larger than 0.90. (b) The key modules were determined from PPI network by MCODE tool (the yellow modules represent the hub genes). (c) Univariate Cox regression analysis with 363 DEGs, listing the top 15 significant factors with *p* < 0.001. (d) Venn plot showing the prognostic-related core genes shared by leading 30 nodes from PPI and top 15 significant factors in univariate Cox. (e) Differential expression of *CCR2* and *P2RY13* in LUAD tumor and normal tissues. (f) Verification of expression of CCR2 and P2RY13 in LUAD tumor and normal tissues by THPA database. TME: Tumor microenvironment; LUAD: Lung adenocarcinoma; PPI network: protein protein interaction network

To further explore the related functions and potential mechanisms of 363 co-expressed genes, we established a PPI network using Cytoscape software with STRING database (Figure 3a) and obtained the top 30 core genes based on the number of nodes (Figure 3b). Meanwhile, 15 prognostic genes were obtained by Cox regression analysis of survival variables in LUAD patients (Figure 3c). Finally, through the intersection analysis of the PPI network and univariate Cox regression, *CCR2* and *P2RY13*, two core genes associated with prognosis, were screened out (Figure 3d). The expression of *CCR2* and *P2RY13* was reduced in tumor tissues (P < 0.05). Among them, *P2RY13* showed a more statistically statistical difference than *CCR2* in normal and tumor tissue. Similar results were observed in the paired analysis of normal and tumor tissues from the same patients (Figure 3e). Also, the expression of *CCR2* and *P2RY13* in LUAD tumor and normal tissues was verified by the THPA database (Figure 3f). After univariate and multivariate Cox regression analysis combined with clinical data, we found that the *P2RY13* gene was still an independent factor affecting the prognosis of LUAD (Table 1).

**Table 1.** Univariate and multivariate cox regression analysis based on LUAD clinicopathological features and *P2RY13* gene

Characteristics	Total(N)	Univariate analysis		Multivariate analysis	
		Hazard ratio (95% CI)	P value	Hazard ratio (95% CI)	P value
T stage (T3&T4 vs. T1&T2)	523	2.317 (1.591–3.375)	**<0.001**	1.804 (1.129–2.882)	**0.014**
N stage (N1&N2&N3 vs. N0)	510	2.601 (1.944–3.480)	**<0.001**	2.072 (1.411–3.043)	**<0.001**
M stage (M1 vs. M0)	377	2.136 (1.248–3.653)	**0.006**	1.235 (0.652–2.341)	0.517
Age (>65 vs. ≤65)	516	1.223 (0.916–1.635)	0.172		
Gender (Male vs. Female)	526	1.070 (0.803–1.426)	0.642		
Smoker (No vs. Yes)	512	1.119 (0.742–1.688)	0.591		
Pathologic stage (Stage III&Stage IV vs. Stage I&Stage II)	518	2.664 (1.960–3.621)	**<0.001**	1.353 (0.831–2.204)	0.224
*P2RY13* (High vs. Low)	526	0.607 (0.452–0.815)	**<0.001**	0.664 (0.470–0.939)	**0.021**

### Analysis of correlation between P2RY13 and prognosis and clinicopathological features of patients with LUAD

**Figure 4.**
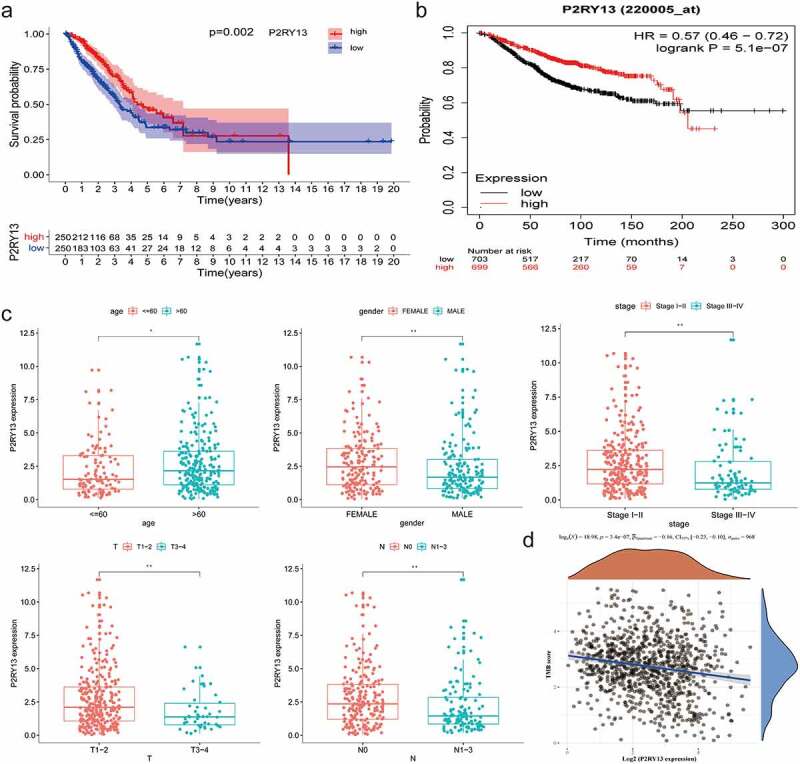
Analysis of correlation between P2RY13 and prognosis and clinicopathological features of patients with LUAD. (a) Kaplan–Meier survival curve for LUAD patients with different *P2RY13* expression. (*P* = 0.002 by log-rank test). (b) The Kaplan-Meier online database was used to analyze the survival of 699 LUAD patients with high *P2RY13* expression and 703 patients with low *P2RY13* expression. (c) The Correlation between *P2RY13* expression and clinicopathological characteristics of LUAD patients (*P < 0.05, **P < 0.01, and ***P < 0.001, with Kruskal–Wallis rank sum test). (d) Spearman’s correlation analysis was used to describe the correlation between *P2RY13* gene expression and TMB(**r = −0.16**). LUAD: Lung adenocarcinoma; TMB: Tumor Mutational Burden

**Figure 5.**
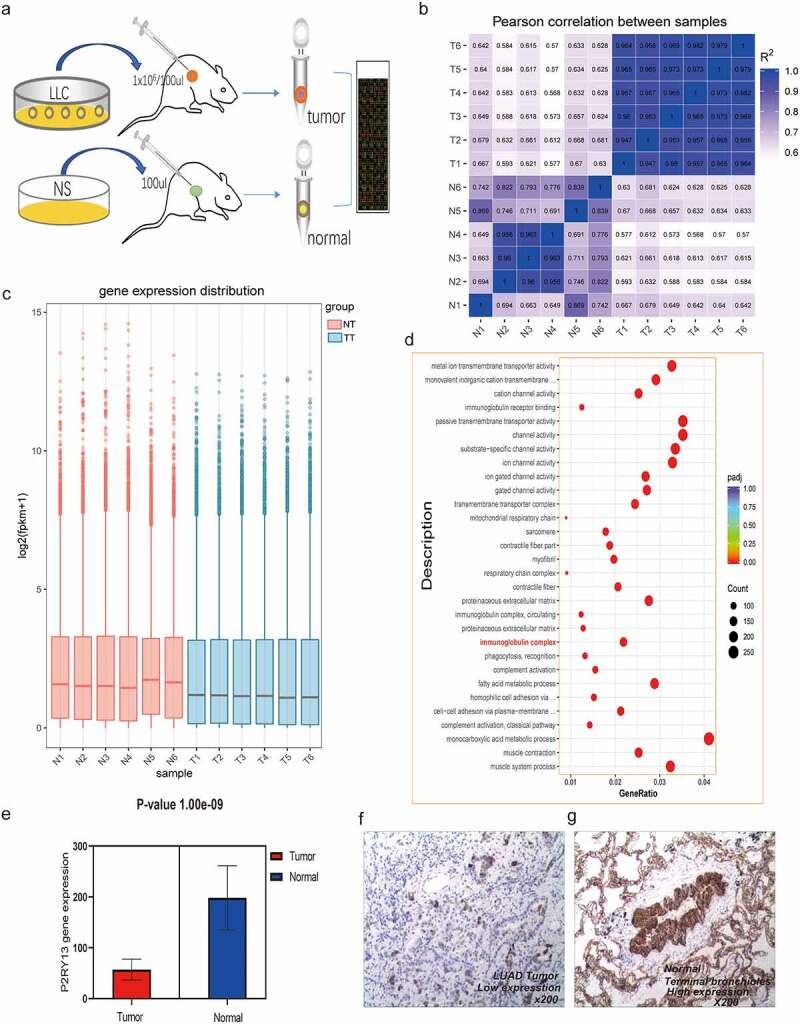
Analysis of RNA-seq results based on C57BL/6 tumor-bearing model and P2RY13 verification. (a) Hematic diagram of the RNA-seq process in this study. (b) Pearson correlation analysis based on gene expression in tumor-bearing model and control group. As shown in the figure, the correlation between tumor models and normal tissues was relatively lower, and the differences between groups based on gene expression were more obvious. (c) The histogram of gene expression distribution in tumor-bearing model group and control group, the gene expression gene of tumor group is lower than that of normal group. (d) Enrichment analysis (KEGG) bubble chart based on DEGs between the two groups. As shown in the figure, DEGs were significantly enriched in pathways related to the immune process. (e) Differential expression of *P2RY13* gene in C57BL/6 lung cancer tissues and normal tissues, the expression of *P2RY13* in tumor tissues was significantly reduced (P < 0.001). (f-g) Expression of P2RY13 protein in human LUAD and normal alveolar tissue (X200, SP). LUAD: Lung adenocarcinoma; KEGG: Kyoto Encyclopedia of Genes and Genomes; DEGs: differentially expressed genes

For the prognostic core gene *P2RY13*, we focused on the correlation between *P2RY13* and the prognosis and clinicopathological features of patients with LUAD. It was shown that the highly expressed *P2RY13* gene group significantly improved prognosis compared to the low expression group (p = 0.002) (Figure 4a). We also analyzed the survival of 699 LUAD patients with *P2RY13* high expression and 703 patients with *P2RY13* low expression with the Kaplan-Meier Plotter online database, and the results were consistent with our research (Figure 4b). In addition, correlation analysis between *P2RY13* and clinicopathological characteristics showed that *P2RY13* gene expression was significantly low in male, young patients, and clinically advanced patients, especially in the T and N states (Figure 4c, Figure S4). We used Spearman’s correlation analysis to characterize the correlation between *P2RY13* gene expression and tumor mutation burden (TMB), which showed a significant negative correlation between them (Figure 4d, r = −0.16). In this study, exponential growth apparatus LLC cells (1X10^6^/100ul) and NS (100ul) were injected into the right rib of C57 mice using microsyringes and sampled for RNA-seq (Figure 5a) On the 17th day after tumor implantation, we performed Pearson correlation coefficient analysis on the tumor-bearing model group and normal tissue group samples based on the RNA-seq data. As shown in the figure, the correlation between gene expression in tumor samples and normal tissues was significantly lower compared to the same group (Figure 5b). Besides, it could be seen from the baseline distribution histograms of the two groups of gene expression that the expression of down-regulated genes in DEGs increased in tumor tissues (Figure 5c). Moreover, we analyzed the KEGG pathway enrichment of DEGs between tumor-bearing model and normal tissue. Interestingly, the immune process-related pathway was significantly enriched (Figure 5d).To further clarify the correlation between *P2RY13* gene expression and the occurrence or development of lung cancer, we verified it from two levels: RNA-seq in mouse models and IHC in human LUAD tissue. Based on the RNA-seq analysis of lung cancer tissue and normal tissue of tumor-bearing mice C57BL/6, we found that the expression of the *P2RY13* gene in tumor tissues was significantly reduced (p < 0.001), and the difference was statistically significant. Importantly, the expression difference of *P2RY13* also showed the same result in human LUAD tissue and normal tissue (Figure 5e-g).

### P2RY13 may have a certain correlation and evaluation significance with the TME of LUAD

**Figure 6.**
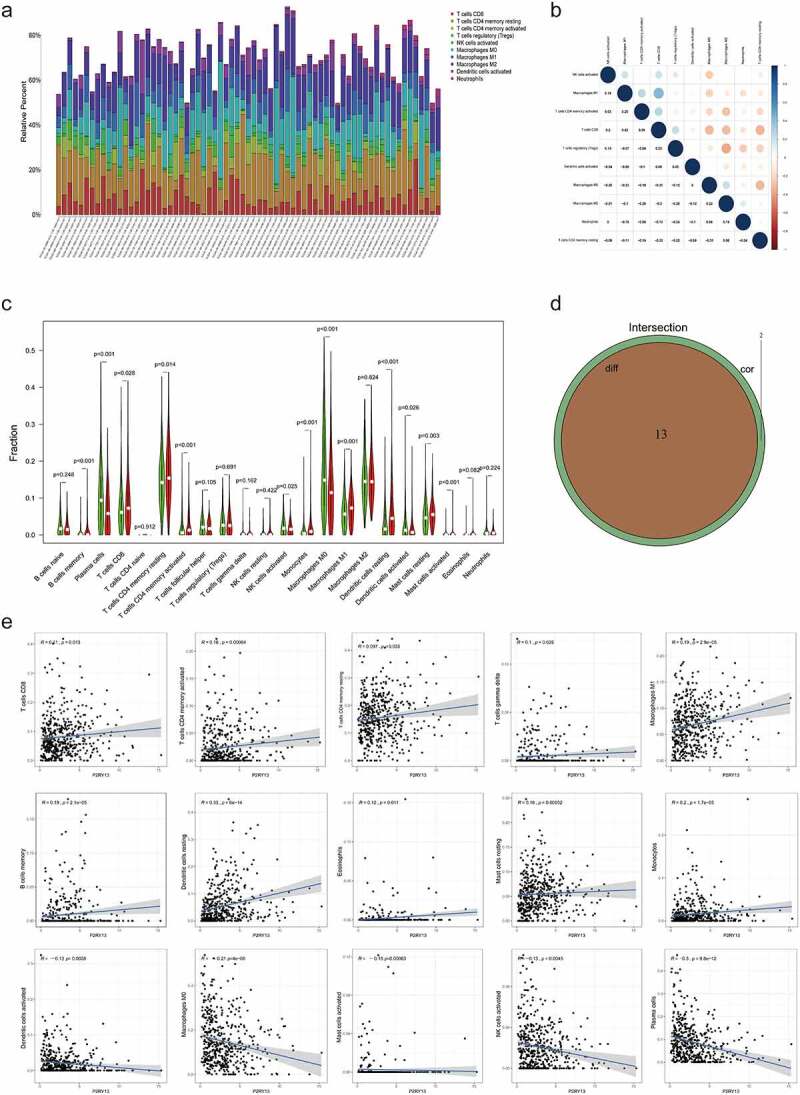
The expression profile of main TICs in LUAD tissues and the difference and correlation analysis with P2RY13 expression. (a) Barplot showing the proportion of main kinds of TICs in LUAD tumor samples. (b) Heatmap showing the correlation between the main TICs and numeric in each tiny box indicating the *p* value of correlation between two kinds of cells. (c) The violin plot showed the difference in the proportion of main TICs in the *P2RY13* high expression and low expression groups. (d) Venn plot showed 13 kinds of TICs related to the expression of *P2RY13* by correlation and difference analysis displayed in violin and scatter plots, respectively. (e) Analysis of the correlation between *P2RY13* and 13 immune cells, which included eight TICs that were positively correlated with *P2RY13* expression and five TICs that were negatively correlated with *P2RY13* expression. TICs: tumor-infiltrating immune cells; LUAD: Lung adenocarcinoma

**Figure 7.**
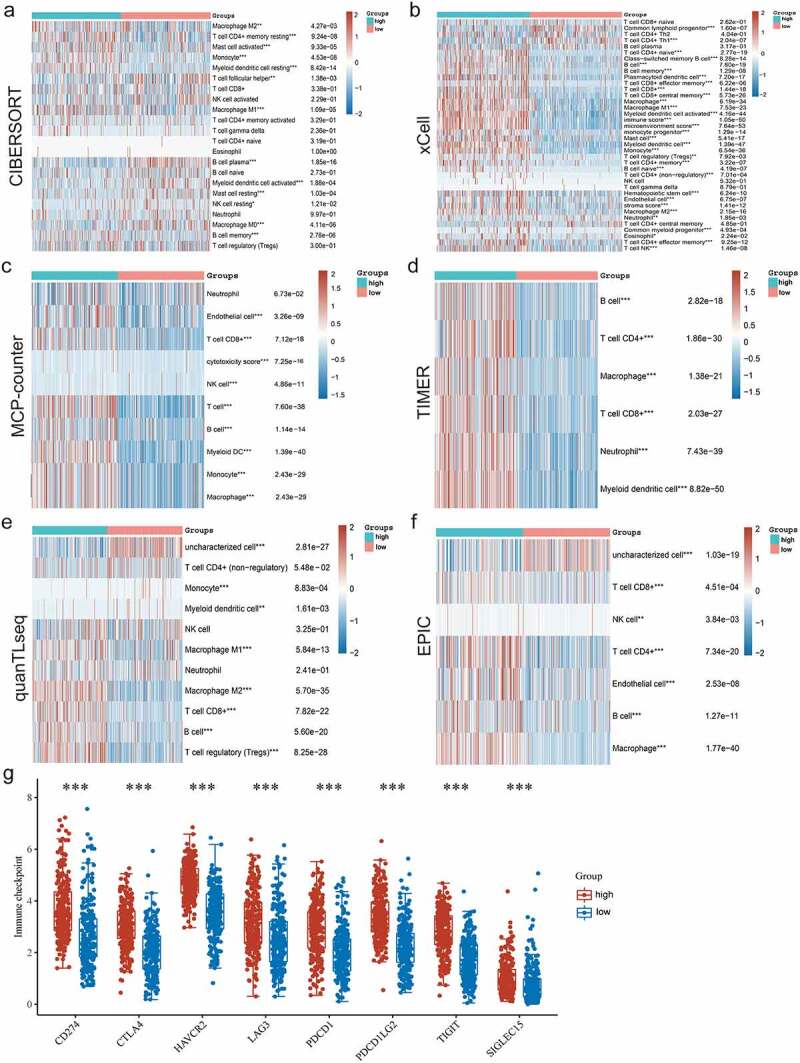
Six different calculation methods with R package were uesd to obtain the violin map and heat maps of P2RY13 gene expression. (a-f) The ‘immunedeconv’ R software package, which integrates six latest algorithms, including TIMER, xCell, MCP-counter, CIBERSORT, EPIC and quanTIseq, were used to verify the correlation between *P2RY13* and immune cells in LUAD, P < 0.05 were selected as the screening condition (*P < 0.05, **P < 0.01, and ***P < 0.001). (g) The differential distribution of immune checkpoint-related genes in *P2RY13* high expression and low expression groups, which were achieved by the R software packages ‘ggplot2’ and ‘pheatmap’. LUAD: Lung adenocarcinoma

**Figure 8.**
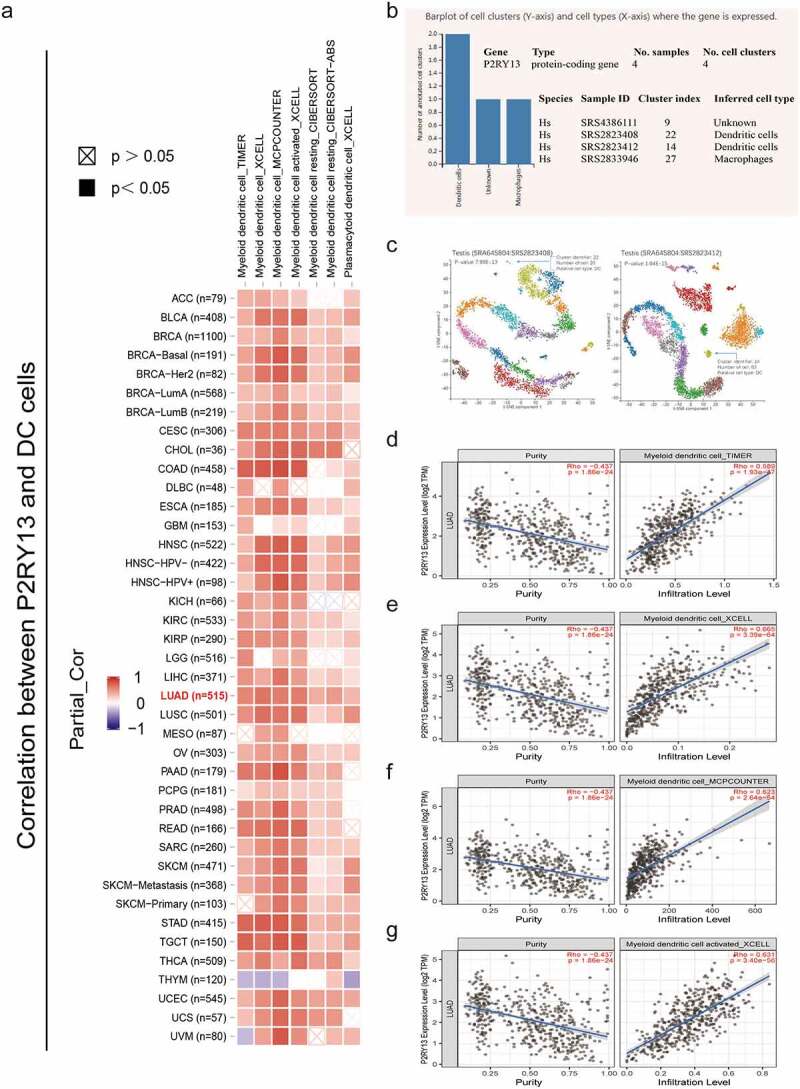
Correlation analysis between P2RY13 expression and immune cell infiltration in pan-cancer tumor tissues. (a) Correlation analysis between *P2RY13* gene expression and DC infiltration in pan-carcinoma based on different algorithms. (b) Screening of immune cells significantly related to *P2RY13* gene expression in PanglaoDB online database based on scRNA-seq, among which DCs were the most significant. (c) Use cluster analysis to visualize the location and number of DC cells in the panorama. (d-g) Correlation analysis between DC and *P2RY13* gene expression in LUAD (r > 0.5). DCs: Dendritic cells; LUAD: Lung adenocarcinoma

To further explore the correlation between the expression of *P2RY13* and TME in LUAD, the proportion of TICs in TME was statistically analyzed by the CIBERSORT algorithm (Figure 6a). In addition, correlation analysis was performed for immune cells mainly involved in immune regulation in TME. Interestingly, CD8^+^T effector cells were significantly positively correlated with M1 macrophages (r = 0.48) and negatively correlated with M2 macrophages (r = −0.3) (Figure 6b). To explore the differences of *P2RY13* gene expression in different immune cells, we obtained violin and heat maps of *P2RY13* gene expression by using six different calculation methods of the R package. After comparative analysis, we found that the expression of *P2RY13* gene in different immune cells was different, especially in 13 lung cancer immune cells (P < 0.05). In addition, the *P2RY13* high expression group was enriched in more immune cells, especially immune effector cells. In contrast, the opposite was true for the *P2RY13* low expression group (Figures 6c, 7a-f). The Venn diagram, obtained from the combined analysis of correlation and difference, showed that 13 kinds of immune cells with *P2RY13* expression differences were correlated with each other (Figure 6d), which included eight TICs that were positively correlated with *P2RY13* expression and five TICs that were negatively correlated with *P2RY13* expression (Figure 6e). Moreover, based on the expression analysis of the transcript genes of eight immune checkpoints in different expression groups of *P2RY13*, tissues with high expression of *P2RY13* were accompanied by higher immune checkpoint distribution (Figure 7g). To further clarify the correlation between the *P2RY13* gene and immune cell infiltration in the TME, scRNA-seq online analysis database PanglaoDB was used to unearth the immune cells that were significantly related to the expression of *P2RY13* in pan-cancer. Four samples with significant expression of *P2RY13* were screened out after statistics, among which dendritic cells (DCs) and macrophages were particularly prominent (Figure 8b). Interestingly, the result was consistent with the previous conclusion. Also, using cluster analysis, the immune cells and mesenchymal cells expressing the *P2RY13* gene were visualized and the number and location of DCs clusters in the TME were marked respectively (Figure 8c, Tables 2–3). Based on this, calculation methods such as TIMER, XCELL, MCPCOUNTER, CIBERSORT, CIBERSORT-ABS were used to further analyze the correlation between the *P2RY13* gene and DCs infiltration in pan-carcinoma (Figure 8a). It can be seen that the expression of *P2RY13* gene was positively correlated with the infiltration of DCs in a variety of tumor tissues in pan-carcinoma. We visualized the data with correlation coefficient R > 0.5 in LUAD, among which TIMER, XCELL, and MCPCOUNTER were particularly significant (Figure 8d-g).

**Table 2.** Cluster analysis of *P2RY13* gene-related cells in SRS2823408 sample based on scRNA-seq

Cluster ID	Number of cells	Inferred cell type	P-value	Adjusted p-value	Activity score
0	452	Germ cells	2.26E-19	8.42E-17	50.0991
1	336	Germ cells	1.64E-31	8.38E-29	121.559
2	311	Germ cells	3.17E-28	1.44E-25	113.027
3	311	Germ cells	8.96E-23	3.67E-20	90.8209
4	296	Germ cells	0.00E+00	0.00E+00	215.656
5	246	Germ cells	1.46E-15	4.27E-13	52.2369
6	245	Germ cells	3.87E-19	1.32E-16	81.4018
7	242	Germ cells	2.28E-14	5.84E-12	47.3752
8	208	Germ cells	0.00E+00	0.00E+00	266.382
9	206	Germ cells	0.00E+00	0.00E+00	251.896
10	204	Peritubular myoid cells	3.15E-13	7.59E-11	44.6885
11	179	Germ cells	5.23E-19	1.65E-16	52.3141
12	179	Germ cells	0.00E+00	0.00E+00	169.134
13	178	Germ cells	0.00E+00	0.00E+00	214.908
14	115	Germ cells	1.01E-14	2.76E-12	60.3915
15	88	Germ cells	0.00E+00	0.00E+00	284.875
16	60	Germ cells	0.00E+00	0.00E+00	309.072
17	58	Spermatocytes	2.65E-06	3.01E-04	62.5466
18	48	Peritubular myoid cells	1.13E-04	8.87E-03	31.3932
19	40	Germ cells	1.58E-10	3.22E-08	44.5504
20	40	Peritubular myoid cells	2.22E-05	2.22E-03	39.6026
21	29	Endothelial cells	3.77E-10	6.71E-08	33.1273
**22**	**20**	**Dendritic cells**	**7.99E-13**	**1.82E-10**	**66.7788**

Source name: Adult testis, number of cells: 2502, cell type: Steady-state Spermatogenic cells, GSM2928382, Homo sapiens, scRNA-seq.

**Table 3.** Cluster analysis of *P2RY13* gene-related cells in SRS2823412 sample based on scRNA-seq

Cluster ID	Number of cells	Inferred cell type	P-value	Adjusted p-value	Activity score
0	804	Peritubular myoid cells	1.61E-17	4.79E-15	58.8716
1	719	Germ cells	2.76E-13	6.55E-11	46.8484
2	434	Germ cells	5.71E-41	2.54E-38	131.514
3	365	Germ cells	0.00E+00	0.00E+00	167.251
4	355	Germ cells	1.36E-29	5.39E-27	115.647
5	347	Germ cells	0.00E+00	0.00E+00	245.895
6	318	Germ cells	0.00E+00	0.00E+00	238.862
7	281	Germ cells	0.00E+00	0.00E+00	200.799
8	276	Germ cells	0.00E+00	0.00E+00	202.086
9	228	Germ cells	0.00E+00	0.00E+00	265.608
10	173	Germ cells	0.00E+00	0.00E+00	210.975
11	121	Germ cells	2.91E-26	1.03E-23	111.739
12	119	Germ cells	1.28E-08	1.90E-06	40.3467
13	111	Germ cells	1.04E-15	2.85E-13	77.8898
**14**	**83**	**Dendritic cells**	**2.52E-15**	**6.41E-13**	**71.955**
15	83	Peritubular myoid cells	1.80E-10	3.55E-08	56.0457
16	70	Smooth muscle cells	1.42E-10	2.98E-08	41.9426
17	69	Spermatocytes	9.07E-06	9.79E-04	34.8291
18	65	Endothelial cells	6.11E-18	1.98E-15	43.9593
19	14	Endothelial cells	1.48E-09	2.52E-07	33.2691

Source name: Adult testis, number of cells: 3086, cell type: Sta-Put enriched Spermatids, GSM2928384, Homo sapiens, scRNA-seq.

## Discussion

The survival, proliferation, invasion, and recurrence of tumors are significantly associated with the presence of cancer stem cells (CSCs), and the exploration of CSC-based target genes has thus become an important biomarker for assessing prognosis [24,25]. Moreover, the tumor microenvironment (TME) in which CSCs reside also plays a vital role in tumor formation and invasion, which contribute to maintaining the stemness of tumor cells [26]. It can directly promote vasculature generation, invasion, metastasis, and chronic inflammation. Increasing evidence suggests that external stimuli mediated by the microenvironment play a key role in the survival and drug resistance of tumor cells [27]. Substantial progress has been made in the study of ‘TME-mediated tumor drug resistance’ [28]. Therefore, blocking or mediating TME may become a new key treatment mode to inhibit tumor progression. It is well known that TME can hinder the anti-tumor function of immune cells. A variety of mechanisms mediated by factors produced by tumor and stromal cells, including reduction of immune effector cell infiltration, down-regulation of major histocompatibility complex (MHC) expression, and up-regulation of immunosuppressive signals, can inhibit all stages of the anti-tumor immune response [29]. Therefore, understanding the phenotype of immune cells in TME is essential for understanding the mechanisms of cancer progression and immunotherapy responses. In addition, mediating TME can also improve the effect of radiotherapy and chemotherapy [30,31], provide new potential targets for targeted therapy, and thus help TME remodel and promote TME from tumor-friendly to tumor-suppressive [32]. In this article, our analysis of LUAD transcriptome data also showed that the abundance of immune cells in TME, especially the ratio of immune and stromal components, was significantly related to the clinicopathological features or prognosis of patients. The above results emphasize the importance of exploring the degree of immune cell infiltration in TME for evaluating the prognosis of LUAD and finding potential targets for immunotherapy.

Over the past three decades, immunotherapy has been considered to be one of the most effective treatment strategies against the minimum tumor burden and the small number of tumor cells that can be accessed by circulating immune cells [33]. A growing number of studies have shown that the tumor immune microenvironment, consisting of various immune cells that promote or suppress immunity, is involved in the progression and transformation of LUAD, and shows good predictive and evaluative power for the prognosis of LUAD patients [34]. Several studies have shown that the abundance of tumor-infiltrating lymphocytes (TILs) is significantly correlated with the 5-year survival rate of NSCLC and low preoperative lymphocyte count is considered to be a poor prognostic signal for patients with early NSCLC [35]. Therefore, TILs can be used as an important factor in assessing the prognosis of LUAD [36,37]. However, is it possible to assess the prognostic survival of patients with LUAD according to the immune-related gene system and select the potential targets that can regulate or reflect TILs? With bioinformatics studies, Bi et al found that the decrease of immune gene *BTK* expression was closely related to clinicopathological features (clinical stage and distant metastasis) and poor prognosis, which showed a significant correlation with the reduction of TILs [38]. Based on the signature of immune-related genes, Song et al established markers from the overall level to predict the prognosis of LUAD and reflect its tumor immune microenvironment, which provides a potential new target for immunotherapy [39]. In this study, the selected prognosis-related core gene *P2RY13* was confirmed to be highly consistent with the TILs of LUAD by difference and correlation analysis, which further indicated that the differentially expressed *P2RY13* gene in LUAD immune cells also predicted a significant correlation with immune cell infiltration. Therefore, it is reasonable to believe that *P2RY13* can be used as a potential marker for evaluating the number of lymphocyte infiltration and prognosis in LUAD.

As one of the purinergic receptors, *P2RY13* is a G protein-coupled receptor that recognizes various endogenous nucleotides as ligands [40]. Studies have shown that extracellular nucleotides can regulate the physiological and pathological processes of all organs [41], so purinergic signal transduction is involved in the formation and progression of many diseases including neurological diseases, phenotype fibrosis, and tumors [42]. It was well known that TME contains a variety of cytokines and extracellular adenosine-triphosphate (ATP) adenosine, other triphosphate or diphosphate nucleotides, such as Uridine diphosphate (UDP), which disrupt the cell-to-cell communication in TME, which has become an important way to suppress tumors [43,44]. Therefore, purinergic receptors are considered as promising drug targets for adjuvant therapy of most cancers [45]. The role of *P2RY2, P2RY6*, and *P2RY12* receptors in gastrointestinal tumors has been reported, including involvement in tumor cell proliferation, metabolism, proliferation, apoptosis, and chemotherapy drug resistance [40]. What is more, in the co-expression network analysis, the selected *P2RY13* gene was confirmed to be significantly related to the survival of LUAD patients. However, the value of *P2RY13* gene in evaluating the prognosis and immune regulation of patients with LUAD has not been reported. Therefore, the study on the function of purinergic receptors in LUAD may provide new targets for urgently needed therapeutic strategies, especially immunotherapy.

Immunotherapy mainly kills tumor cells by activating the immune cell components of the TME. Therefore, identifying the anti-tumor active components of the TME is particularly important in immunotherapy [46]. Studies have shown that only 14% to 20% of patients with advanced NSCLC have a durable response to monoclonal antibodies [47]. Therefore, addressing the low response to immunotherapy is the key to solving the dilemma of immunotherapy. Based on the TME, some scholars further divided the immune category into active immunity level and exhausted immunity level [46]. The former exhibited prominent features of IFN, T cells, and M1 macrophages, while the latter showed a fatigued immune response with activation immunosuppressive factors such as TGF-*β* [48]. This is consistent with the conclusion of our study. In the TME enriched with *P2RY13* gene, it was associated with increased infiltration of CD4/CD8 T cells, M1 macrophages, and B cells. In contrast, the low expression of *P2RY13* gene was negatively correlated with immunosuppressive cells and immunosuppressive factors such as TGF-*β*. The above-mentioned shows that we can judge the effect of tumor immunotherapy based on the expression level of the *P2RY13* gene. Furthermore, at the genetic level, we can find a solution for the future breakthrough of the bottleneck problem of immunotherapy. Therefore, understanding the interaction between stromal cells, immune cells and tumors plays an important role in improving the effectiveness of current immunotherapy.

Despite our efforts, this study still has some limitations. This research was based on the screening and analysis of data from public databases. Although we have done some experimental verification, it was limited to the prognostic-related gene or protein expression, and more research based on the molecular mechanism or immune regulation mechanism needs to be implemented. Therefore, it is not possible to conclude that *P2RY13* can be used to comprehensively assess the immune microenvironment and prognosis of LUAD. However, the mutant gene still provides direction or ideas for our subsequent studies.

## Conclusion

It is important to screen for a mutant gene or biomarker that can predict the abundance of immune cell infiltration and prognosis of LUAD. The correlation analysis between *P2RY13* and immune cells obtained based on the six latest algorithms provided an assessment perspective for this. The high expression of *P2RY13* indicated an increase in immune cell infiltration in the TME and a good prognosis. Although sufficient basic experiments and clinical case validation were lacking, it provided a new direction for the development of subsequent related research and clinical treatment.

## Supplementary Material

Supplemental Material

## Data Availability

Please refer to the raw RNA-seq data of C57BL/6 mouse Lewis tumor-bearing model that we uploaded to BioSample database through the following link: https://www.ncbi.nlm.nih.gov/sra/PRJNA752479. For additional raw data, please contact the corresponding author for more information.

## Highlights

1. *P2RY13* was associated with prognosis and clinicopathological features in LUAD.
2. *P2RY13* was enriched in immune-related pathways and down-regulated in LUAD.
3. *P2RY13* was associated with infiltration of major immune cells especially DCs.
