# Supplementary material for: Evaluation of tumor microenvironmental immune regulation and prognostic in lung adenocarcinoma from the perspective of purinergic receptor P2Y13
Source: Bioengineered. 2021 Sep 8;12(1):6286–304. doi: 10.1080/21655979.2021.1971029 (PMC8806861; doi:10.1080/21655979.2021.1971029)
Supplement: Supplemental Material [file KBIE_A_1971029_SM7154.zip › supplementary/Figure Legends.docx]

***Figure S1. Heatmap for DEGs in ImmuneScore and StromalScore.*** Heatmap for DEGs generated by comparison of the high score group vs the low score group in ImmuneScore (Figure S1a) and StromalScore (Figure S1b) by “limma” package with P< 0.05 and fold-change >1 as the cut-offs. DEGs: differentially expressed genes.

***Figure S2. Enrichment analysis of GO for DEGs based on ImmuneScore and StromalScore.*** (a) Barplot of GO enrichment analysis for 363 DEGs. (b) Bubble of GO enrichment analysis for 363 DEGs. (c) Circos of GO enrichment analysis for 363 DEGs. terms with *p* and *q* < 0.05 were believed to be enriched significantly. GO: Gene Ontology; DEGs: differentially expressed genes.

***Figure S3. Enrichment analysis of KEGG for DEGs based on ImmuneScore and StromalScore.*** (a) Barplot of KEGG enrichment analysis for 363 DEGs. (b) Bubble of KEGG enrichment analysis for 363 DEGs. (c) Circos of KEGG enrichment analysis for 363 DEGs. terms with *p* and *q* < 0.05 were believed to be enriched significantly. KEGG: Kyoto Encyclopedia of Genes and Genomes; DEGs: differentially expressed genes.

***Figure S4. Analysis of correlation between P2RY13&CCR2 and clinicopathological features of patients with LUAD.*** (a-f) The Correlation between *P2RY13* expression and clinicopathological characteristics of LUAD patients (with Kruskal–Wallis rank sum test). (g-l) The Correlation between *CCR2* expression and clinicopathological characteristics of LUAD patients (with Kruskal–Wallis rank sum test). LUAD: Lung adenocarcinoma.
