# Supplementary figures and images for: Evaluation of tumor microenvironmental immune regulation and prognostic in lung adenocarcinoma from the perspective of purinergic receptor P2Y13
Source: Bioengineered. 2021 Sep 8;12(1):6286–304. doi: 10.1080/21655979.2021.1971029 (PMC8806861; doi:10.1080/21655979.2021.1971029)

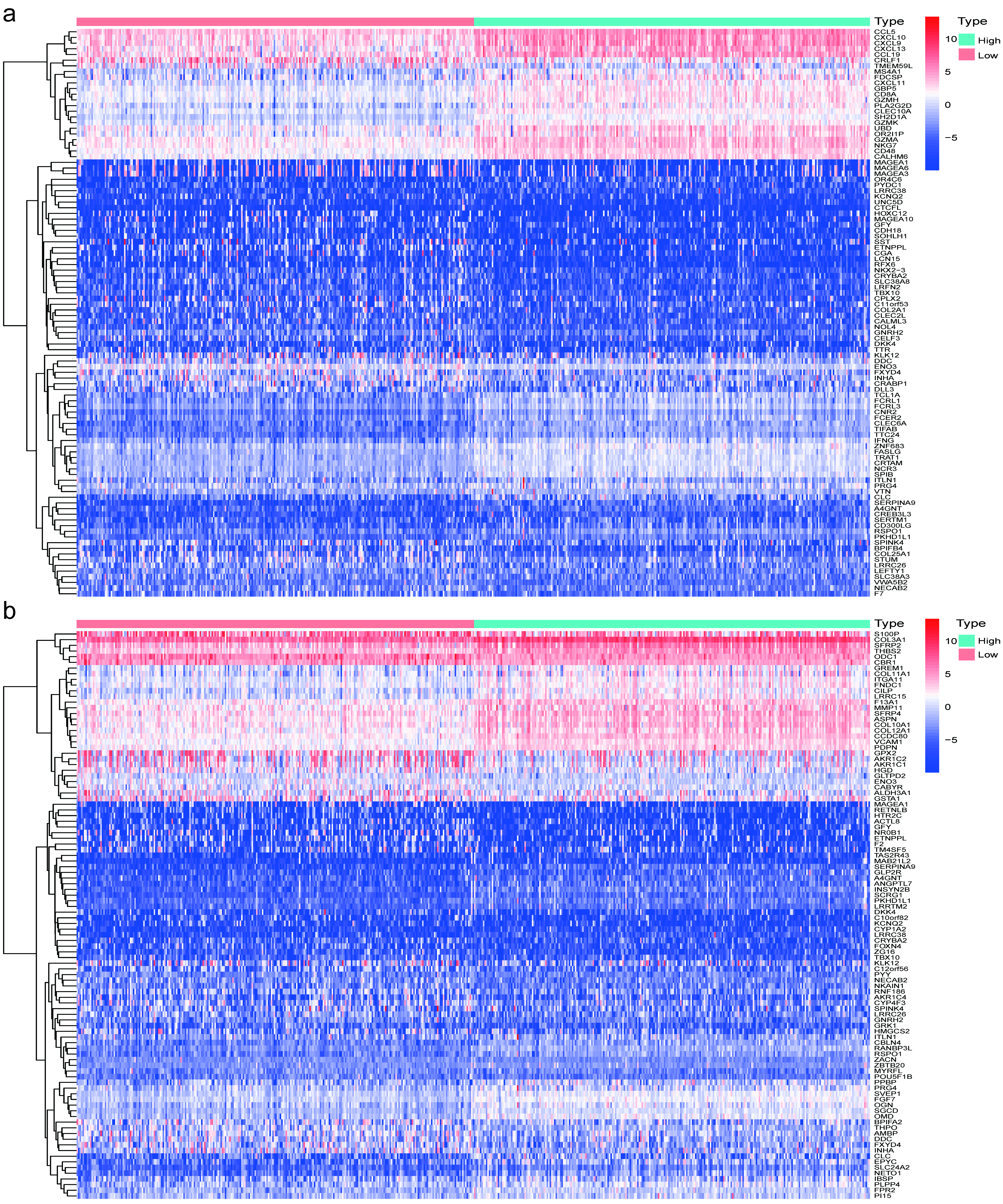

Supplement: Supplemental Material [file KBIE_A_1971029_SM7154.zip › supplementary/Figure S1.tif]

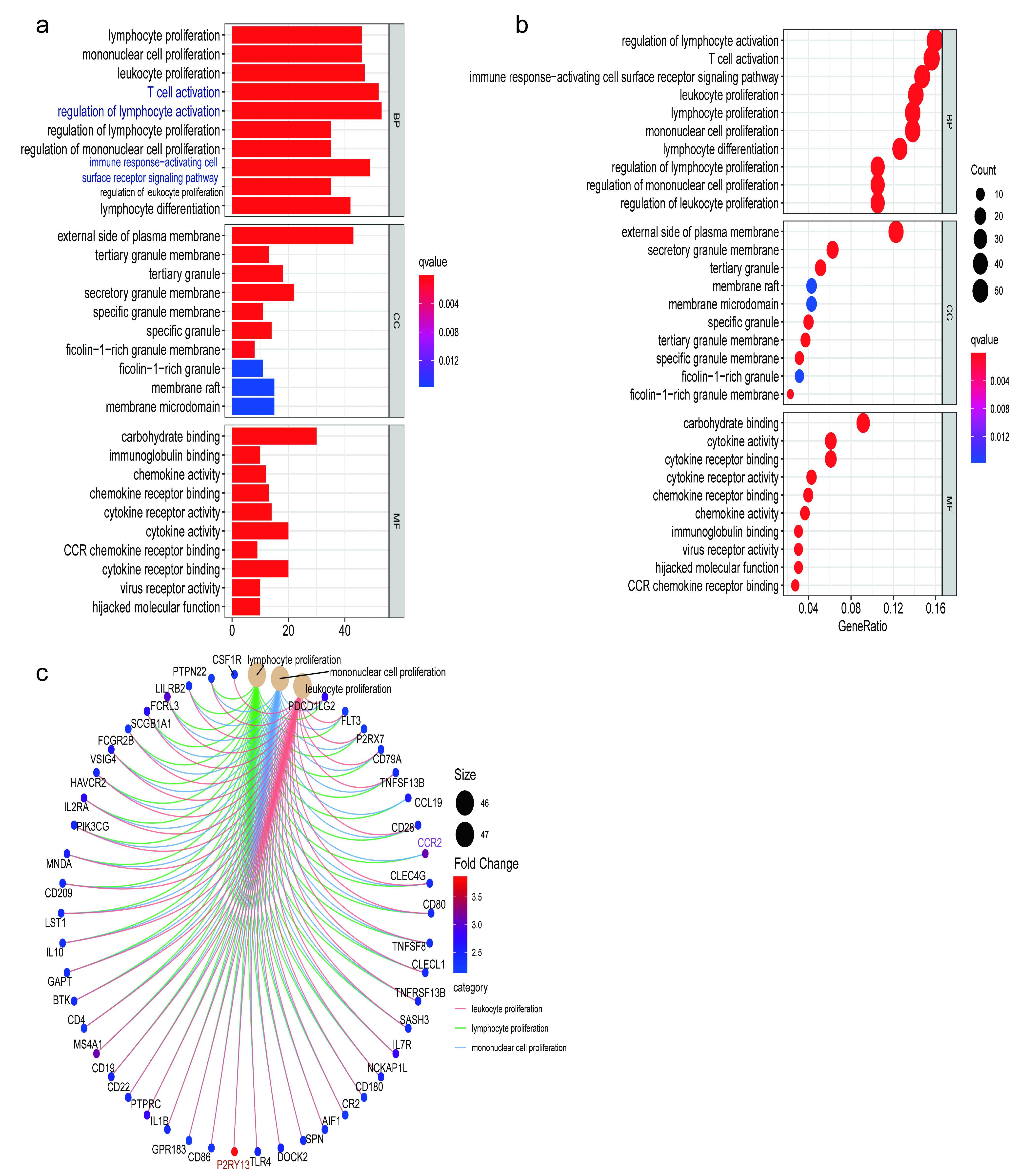

Supplement: Supplemental Material [file KBIE_A_1971029_SM7154.zip › supplementary/Figure S2.tif]

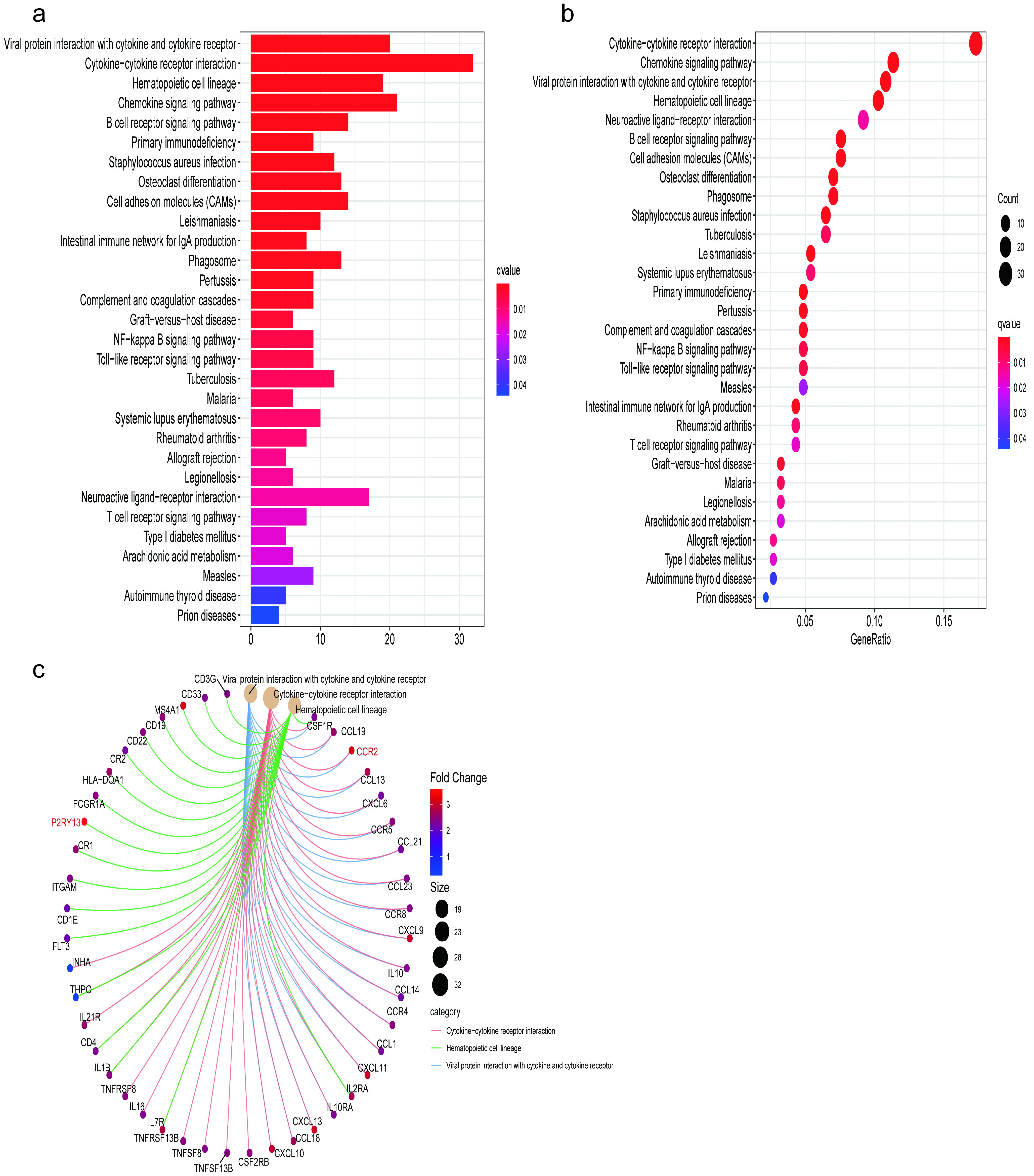

Supplement: Supplemental Material [file KBIE_A_1971029_SM7154.zip › supplementary/Figure S3.tif]

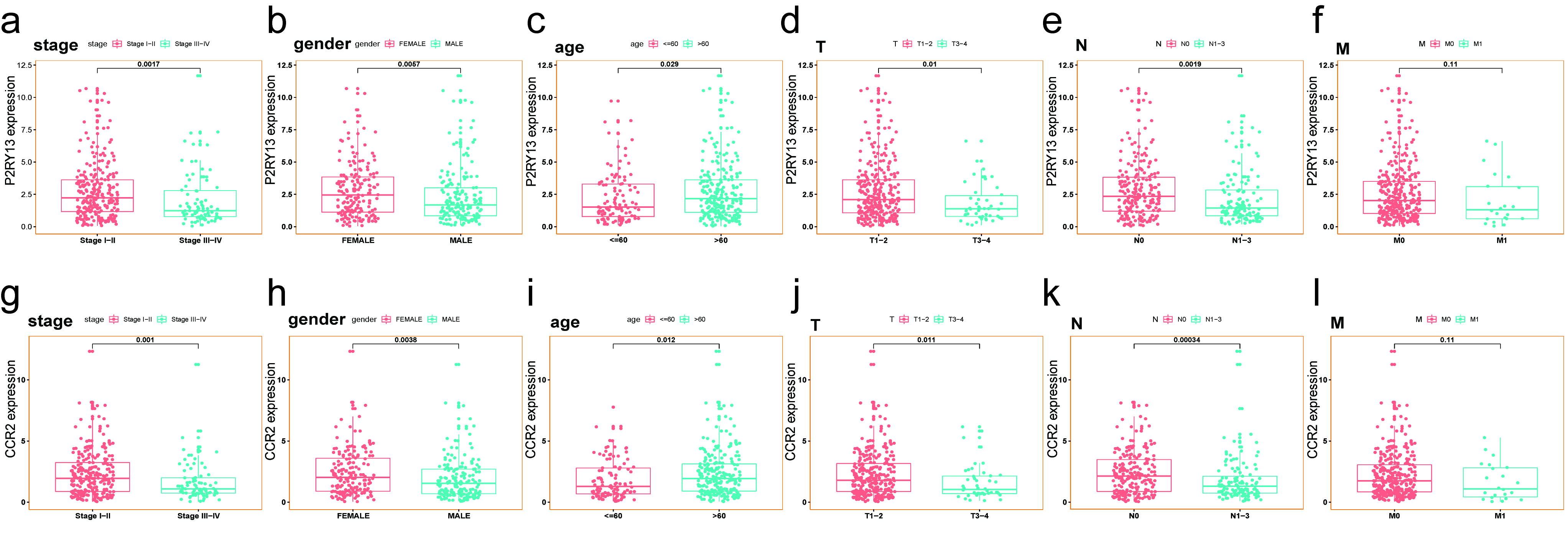

Supplement: Supplemental Material [file KBIE_A_1971029_SM7154.zip › supplementary/Figure S4.tif]
